# The importance of early bonding on the long-term mental health and resilience of children

**DOI:** 10.1080/17571472.2015.1133012

**Published:** 2016-02-24

**Authors:** Robert Winston, Rebecca Chicot

**Affiliations:** ^a^ Science and Society, Imperial College, London, UK; ^b^ The Essential Parent Company, Cambridge, UK

**Keywords:** Child development, mental health, parenting, bonding (psychology), neurodevelopmental disorders, epigenomics

## Abstract

Human babies are born very dependent on their parents. They undergo huge brain development, growth and neuron pruning in the first two years of life. The brain development of infants (as well as their social, emotional and cognitive development) depends on a loving bond or attachment relationship with a primary caregiver, usually a parent. There is increasing evidence from the fields of development psychology, neurobiology and animal epigenetic studies that neglect, parental inconsistency and a lack of love can lead to long-term mental health problems as well as to reduced overall potential and happiness. In this paper, the authors consider the evidence for this claim across several disciplines and conclude that the support of babies and their parents in the first two years of life to be a crucial aim of public health groups in the community.

## Why this matters to me

The evidence on the powerful role of loving nurture in the emotional, social and cognitive development of children is powerful. Parenting is therefore more important than we could ever have imagined. Although I (Robert Winston) have published over 300 papers in medical journals and worked to develop IVF techniques, if I’m really honest, the most important achievement is undoubtedly my own three children. I don’t have any doubt about that. And all of us in different ways are capable of contributing to the next generation both as parents, health care professionals and as a society.

## Key messages

Infancy is a crucial time for brain development. It is vital that babies and their parents are supported during this time to promote attachment. Without a good initial bond, children are less likely to grow up to become happy, independent and resilient adults.

## The science of epigenetics

Imagine if the hugs, lullabies and smiles from parents could inoculate babies against heartbreak, adolescent angst and even help them pass their exams decades later. Well, evidence from the new branch of science called epigenetics is reporting that this long-term emotional inoculation might be possible.

The human brain is an amazing organ made up of over 100 billion brain cells that each connect to over 7000 other brain cells.[1] It’s more complicated than a computer, in fact it’s most complicated object in the known universe.

The most important stage for brain development is the beginning of life, starting in the womb and then the first year of life. By the age of three, a child’s brain has reached almost 90% of its adult size.[2] This rapid brain growth and circuitry have been estimated at an astounding rate of 700–1000 synapse connections per second in this period.[3] The experiences a baby has with her caregivers are crucial to this early wiring and pruning and enable millions and millions of new connections in the brain to be made. Repeated interactions and communication lead to pathways being laid down that help memories and relationships form and learning and logic to develop.[4] This means a human baby’s brain is both complicated and vulnerable.[5]

## Use it or lose it

If positive experiences do not happen, the pathways needed for normal human experiences may be lost. This is often referred to as the ‘use it or lose it’ principle.[5] Tragic case studies of ‘feral’ children who have survived with minimal human contact illustrate the severe lack of language and emotional development in the absence of love, language and attention. In the same way, even though babies have a deep genetic predisposition to bond to a loving parent, this can be disrupted if a baby’s parents or caregivers are neglectful and inconsistent.

Indeed longitudinal studies have reported that a child’s ability to form and maintain healthy relationships throughout life may be significantly impaired by having an insecure attachment to a primary caregiver.[6]

Teicher [7] has reported the following pathology in children who suffered neglect (an extreme form of insecure attachment) in their early years

• Reduced growth in the left hemisphere which may lead to associated increased depression risk for depression.
• Increased sensitivity in the limbic system which can lead to anxiety disorders.
• Reduced growth in the hippocampus that could contribute to learning and memory impairments.

These findings have been backed up by cases of extreme neglect and outcomes of children raised in Romanian orphanages. Rutter et al. [8] studied the development of children adopted from Romanian orphanages who were adopted into loving families at different ages. When each child was 6 years old, the researchers assessed what proportion of these adopted children was functioning ‘normally’. They found that 69% of the children adopted before the age of 6 months; 43% of the children adopted between the ages of 7 months and 2 years and only 22% of the children adopted between the ages of 2 years and 3½ years were functioning normally.

## The most valuable thing is love

This highlights the importance of supporting parents and babies in their crucial early years. However, parents can worry about things that just aren’t important to their children’s brain development and well-being such as giving them their own room, buying them toys and taking them on expensive holidays. Instead, the most valuable gift that a child can receive is free; it’s simply a parent’s love, time and support. This is no empty sentiment; science is now showing why baby’s brains need love more than anything else.

The new science of epigenetics is discovering more and more how our genes and our brains are affected by the lives we lead. For example, Champagne et al. [9] showed that (related and unrelated) mice put in the care of loving mothers (who are attentive and lick them caringly) grow up to be better mothers themselves when they have pups. This effect is so strong that it can even stretch over two generations, with granddaughter mice being better mothers and be able to cope with stress better too, all because their grandmother took good care of their mother. These long-lasting benefits of good parenting in mice are dependent on chemical changes in the DNA of the mice.

These same staggering effects (called ‘methylation changes’) on the brains of mice have also now been found in humans. Studies on the brains of people who committed suicide and were abused as children show the same sorts of chemical patterns as neglected mice.[10]

## Implications for health care

If depriving infants of a loving family environment causes lasting damage to their emotional well-being, their intelligence and their capacity to develop fully, what are the implications for public health in the 21st century? Being a parent has changed radically from the way human beings have had families over the last 50,000 years. Expectant parents today have very little practical experience of babies in modern society.

For tens of thousands of years, new parents would have spent many years in extended families learning the skills of parenthood by osmosis from their parents, grandparents, aunts, older siblings, cousins as well as having responsibilities for their own younger brothers and sisters. Today, few parents get this opportunity to be immersed in early family life as extended families. Living in close proximity is largely a thing of the past in the UK.

A first-time pregnant woman today often only has her pregnancy (a mere nine months) to prepare for being a parent. They can therefore be hit hard by the shock of being a new parent and feel very unconfident about how to bond and care for their baby. Post-natal mood disorders are common and a potential barrier to bonding and optimal development of newborns.

A 2012 study by the Essential Parent Company showed that around 80% of new parents felt both anxious and completely unprepared with the practical skills they need to look after their new baby.[11] The UK has one of the lowest breastfeeding rates in Europe which is perhaps not surprising when the survey reported a third of UK parents asked had never seen a family member breastfeeding. Breastfeeding is a learned skill and without seeing it happening, lots of new mums really struggle to know what to do, and usually leave hospital before their milk has come in and breastfeeding has been established. This can lead to disappointment, sadness and stress for the mum and means that by six weeks of age only 20% of British babies are still being breastfed.

These vital practical and loving parenting skills are the building blocks of babies’ care and well-being. There are few things, in our mind, that are more important to the future of our society than understanding the importance of a well-attached baby and seeking to support infants and their parents in the community.

Indeed we are in agreement with Dr Jack Shonkoff, the Director of the Center on the Developing Child at Harvard University [12] and adviser to UNICEF. He argues for a ‘*new role for biology*’ in early year’s provision and policy in focussing interventions and support for parents’ needs for emotional and practical support as a way in to promoting secure attachment and early resilience in children.

To this end, how can health care professionals help practically in the community?

In the antenatal period, a pregnant woman is very open to new information as she prepares to be a mother. We recommend classes and baby care videos to build practical skills that help mothers to bond with their unborn baby. After birth they can continue this learning through experience – skin-to-skin contact, early breastfeeding, cuddling and carrying the baby. They need to have plenty of time in face-to-face contact to promote non-verbal communication and chatting with the baby.

The ‘Well Baby Clinic’ is a great community space to support new parents and babies. There are so many simple tips that can be shared in their space that can build parents’ confidence and happiness such as encouraging lots of eye contact, lots of cuddling and sharing books (from an early age) as all these activities help to promote bonding. Depressed parents can feel like their baby ‘hates’ them or thinks they are a terrible parent. Again, health care professionals can use this time to reassure parents that babies need very simple interactive things – cuddles, responsiveness, smiling and chatting. When parents understand that their babies are not capable of judging them they can feel reassured and confident, knowing that their baby is totally open to loving them and that s/he prefers their voice and their skin to those of anyone else.

Health care professional can share this basic and reassuring information in everyday, one-to-one conversations e.g. as they weigh babies, and also in simple leaflets and posters that promote a warm and gentle approach to parenting and to themselves.

## Disclosure statement

The *Essential Parent Company* is a small private company. The visual materials we have produced were funded by four ‘angel investors’ who allow us to offer independent, evidence-based advice to parents and health care professionals. We also work with charities and expert organisations to ensure that our videos and articles are independent and evidence based.

## Acknowledgements

We would like to thank our long-term partners UNICEF UK Baby Friendly, The Royal College of Paediatrics and Child Health, St John’s Ambulance, The Child Accident Protection Trust as well as the following groups who have used our materials and helped us to reach and support families around the UK; Barnardo’s, Save the Children, NCT and PACT. Finally, we would like to thank colleagues from the Infant Mental Health Foundation for working to educate health care professionals and public health officials on the importance of early attachment relationships in the development and mental health of children and adults.
